# Tropical Rainforest Restoration Plantations Are Slow to Restore the Soil Biological and Organic Carbon Characteristics of Old Growth Rainforest

**DOI:** 10.1007/s00248-019-01414-7

**Published:** 2019-08-01

**Authors:** Mark T. L. Bonner, Diane E. Allen, Richard Brackin, Tim E. Smith, Tom Lewis, Luke P. Shoo, Susanne Schmidt

**Affiliations:** 1 School of Agriculture and Food Science, University of Queensland, Brisbane, Queensland 4072 Australia; 2 Department of Forest Ecology and Management, Swedish University of Agricultural Sciences, 90736 Umeå, Sweden; 3 Department of Environment and Science, Brisbane, Queensland 4001 Australia; 4 Department of Agriculture and Fisheries, Queensland Government, University of the Sunshine Coast, Sippy Downs, 4556 Australia; 5 Faculty of Science, Health, Education and Engineering, University of the Sunshine Coast, Sippy Downs, 4556 Australia; 6 School of Biological Sciences, University of Queensland, Brisbane, Queensland 4072 Australia

**Keywords:** Mixed-species plantations, Soil fungi and bacteria, Soil carbon sequestration, Microbial function and composition, Microbial ecology, Land use change

## Abstract

Widespread and continuing losses of tropical old-growth forests imperil global biodiversity and alter global carbon (C) cycling. Soil organic carbon (SOC) typically declines with land use change from old-growth forest, but the underlying mechanisms are poorly understood. Ecological restoration plantations offer an established means of restoring aboveground biomass, structure and diversity of forests, but their capacity to recover the soil microbial community and SOC is unknown due to limited empirical data and consensus on the mechanisms of SOC formation. Here, we examine soil microbial community response and SOC in tropical rainforest restoration plantings, comparing them with the original old-growth forest and the previous land use (pasture). Two decades post-reforestation, we found a statistically significant but small increase in SOC in the fast-turnover particulate C fraction. Although the δ^13^C signature of the more stable humic organic C (HOC) fraction indicated a significant compositional turnover in reforested soils, from C_4_ pasture-derived C to C_3_ forest-derived C, this did not translate to HOC gains compared with the pasture baseline. Matched old-growth rainforest soils had significantly higher concentrations of HOC than pasture and reforested soils, and soil microbial enzyme efficiency and the ratio of gram-positive to gram-negative bacteria followed the same pattern. Restoration plantings had unique soil microbial composition and function, distinct from baseline pasture but not converging on target old growth rainforest within the examined timeframe. Our results suggest that tropical reforestation efforts could benefit from management interventions beyond re-establishing tree cover to realize the ambition of early recovery of soil microbial communities and stable SOC.

**Electronic supplementary material:**

The online version of this article (10.1007/s00248-019-01414-7) contains supplementary material, which is available to authorized users.

## Introduction

Globally, the extent of old-growth forests is shrinking and the area of human-altered and secondary forests is increasing, with the changes particularly rapid in the tropics [1]. Global carbon (C) cycling and biodiversity are perturbed by the changes in forest cover [2, 3], but the extent of perturbation remains under debate, largely because of contention over the capacity of plantations and secondary forests for C sequestration and biodiversity conservation [3–5]. Belowground processes remain poorly studied and are a major knowledge gap that hinders the holistic understanding of the current and future value of secondary forests. Land use change from old-growth to other types of forest appears to consistently catalyse soil organic matter (SOM) loss [6, 7], compromising soil structure, fertility and C sequestration [8, 9].

Ecological restoration plantations appear to balance aboveground biodiversity and C sequestration goals, often demonstrating a strong capacity for reinstating aboveground rainforest structure and species richness within a few decades of establishment [5, 10–12], although floristic composition is difficult to recover even when planting many locally native tree species at high density [5, 12]. The efficacy of ecological plantings for soil restoration is less understood. No consistent pattern for SOM recovery in the first few decades post-planting has emerged [13–17], although soil structure may change rapidly [13, 18]. Recovery of soil microbial communities through forest restoration is particularly poorly studied [but see 19], with claims of high microbial plasticity and functional redundancy allowing rapid functional response to land use change [20, 21] conflicting with evidence of legacy effects of former land use on soil microbial traits many decades on [22, 23].

Poor understanding of soil microbiological responses to forest restoration is not only a knowledge gap from the perspective of biodiversity conservation—forest soils are among the most species diverse systems on Earth [24]—but substantially obfuscates the potential for recovery of SOM and related ecosystem services from investment in forest restoration. Soil microbes are responsible for decomposition of SOM [25], their biomass and residues are primary chemical precursors of SOM [26–30], and soil microbial community composition may affect the fraction of plant litter that becomes microbial SOM precursor material [31]. A compelling argument can therefore be made that soil microbial traits and SOM formation are strongly coupled, but whole-system benefits of investment in active forest restoration cannot be accurately anticipated because of the paucity of empirical study of soil biological, chemical and physical responses to ecological restoration planting.

Agroecosystems generally harbour lower SOM levels than forest ecosystems [6], but the mechanisms for SOM loss with conversion to agriculture are disputed. Well-supported hypotheses for SOM losses in agricultural soils include soil disturbance through tillage, lower quantities and altered chemical composition of plant residues and use of inorganic fertilizers [6, 32–34]. However, recent work [29, 35] has laid the foundations for a working model that explains patterns of SOM under agriculture and forests. The model predicts that simultaneous supply of labile substrates, which can be metabolized comparatively efficiently, and of diverse and recalcitrant plant litters, which provide conditions that favour a more efficient microbial community [36, 37], minimizes C loss via respiration and increases supply of microbe-derived SOM precursor compounds. The model explains low SOM levels under agricultural land uses as primarily caused by (i) soil disturbance subjecting more soil C to decomposition and erosion while disfavouring microbes that are slow-growing, oligotrophic and metabolically efficient [38], and (ii) the presence of plant residues that are chemically too homogeneous to promote a functionally diverse microbial community. The model yields the management recommendation that increasing SOM under conditions of low soil disturbance will depend on maximizing soil microbial functional diversity, through maximizing the phylogenetic and/or functional diversity of plant litter.

Here, we investigate soil under mixed species plantings (4–34 years since planting) and evaluate recovery relative to reference soils under pasture and rainforest. Specifically, we assess the applicability of the abovementioned working model of SOM formation in the tropics, and examine the efficacy of restoration plantings for reinstating microbial composition and function. The choice of pasture as an agricultural soil eliminates the potentially confounding factors of tillage and low litter inputs, allowing a targeted evaluation of the interplay between land use, microbial community, and SOM. Further, we targeted pastures that were not subject to regular application of inorganic fertilizers and were actively grazed at a low to moderate intensity. We hypothesised that SOM and microbial efficiency would be highest under remnant rainforest, lowest under pasture and intermediate under restoration plantings.

## Materials and Methods

### Study Sites

The study design compared soils from under mixed species plantings and two reference conditions: pastures (representing the baseline prior to reforestation) and rainforest (representing the baseline prior to clearing for pasture, as well as a hypothetical endpoint of reforestation). All sites were located across uplands in tropical north-eastern Australia (16.56–17.43 S, 145.36–145.65 E). The study region consists of a mosaic of pasture, cropland and small patches of plantations, secondary forest and remnant complex notophyll and mesophyll forest [5].

Nineteen 0.3-ha plots distributed across eight sites were sampled for soil microbial traits and SOM content. Each site consisted of two matched plots: a reference pasture and a mixed species planting. Three sites also included a matched plot in reference rainforest. All but two of the pasture sites were actively grazed at a low to moderate intensity, and consisted largely of the grass species *Urochloa decumbens* (Stapf) R.D.Webster. Reference rainforest sites had a closed canopy > 25 m in height and a high diversity of structural features, life-forms and tree species. The mixed species plantings were established by landholders or regional land care groups using a high diversity (20 species or more) of native tree seedlings in excess of 1000 stems per hectare and ongoing weeding until canopy closure (about 3–4 years) (landholders, personal communication), akin to the ‘environmental restoration plantings’ described by Kanowski and Catterall [10]. Plantings were excluded from grazing since establishment and ranged in age from 4 years old to 34 years old, with a mean of approximately 17 years (Fig. S1).

**Fig. 1:**
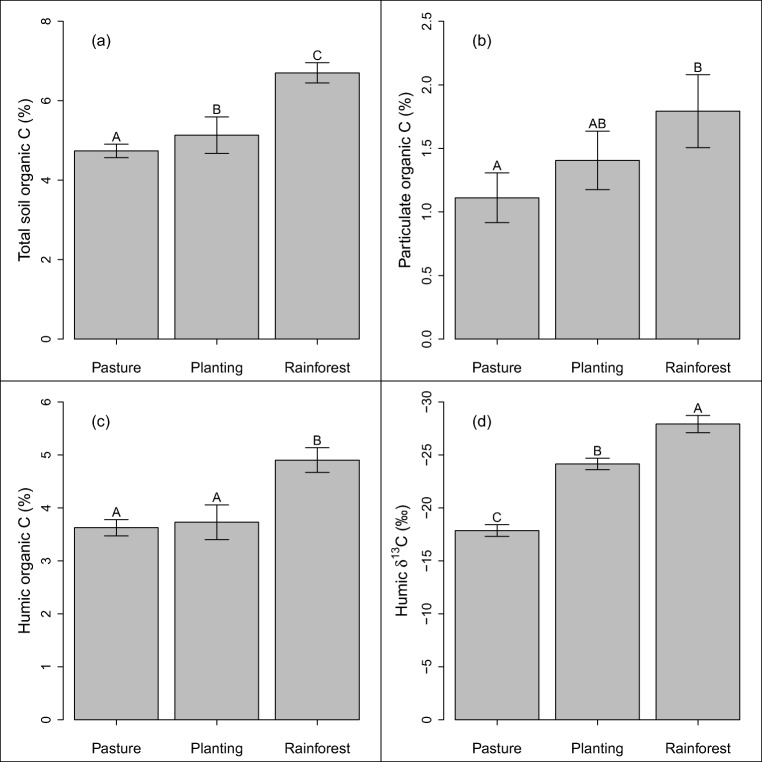
Topsoil (0–10 cm depth) carbon (C) characteristics of pasture, mixed species rainforest restoration plantings and reference rainforest sites in tropical north-eastern Australia. The first three panels depict organic C content (%) associated with **a** the sum of all organic forms of C present in ≤ 2 mm soil, **b** the coarse (particulate) fraction (50–2000 μm particles) and **c** organic C associated with the fine (humus) fraction (≤ 50 μm particles). The fourth panel depicts **d** δ^13^C of humic organic C HOC (‰) with particle size below 50 μm. Error bars for plantings represent standard error of the mean, while errors bars for the two other land uses represent standard errors of difference from revegetation (see the ‘Statistics’ subsection of the ‘Materials and Methods’ section for more detail). Letters above bars represent Tukey’s honest significant differences

The eight sites were selected from a larger pool of candidate sites with the criteria of ensuring that plots in the contrasting land uses were well matched within each site for aspect, slope, soil type to two metres depth, and land use history, which typically was at least 30 years of active pasture since original forest clearing. Pastures were fertilized upon establishment but were not subject to regular fertilization thereafter (landholders, personal communication). Across the study sites, elevation ranged from 600 to 1000 m, mean annual temperature minima and maxima ranged from 14.4 to 15.6 °C and 25.3 to 26.0 °C respectively, and mean annual precipitation ranged from approximately 1400 to 2000 mm (station numbers 031034, 031193, 031029, 031184, 031183, Australian Bureau of Meteorology). The dominant soil types sampled were acidic Rhodic Ferralsols and Dystric Cambisols (FAO soil classification system), with pH values ranging from 4.2 to 6.5.

### Soil Assessment

For microbial analyses, topsoil cores were collected from nine evenly spaced locations in each plot, which were subsequently pooled into three bulked samples per plot. These samples were kept field-moist for 2 weeks to ameliorate confounding effects of labile C [39] before acclimation in soil microcosms for 3 days at 27 °C, 90% humidity and 60% water holding capacity, in order to control for variations in soil microclimate conditions between land uses. This involved 40–45 g soil placed unsieved into microcosms constructed from 50-mL centrifuge tubes [40] and incubated in the dark (Clayson Incubator, Clayson Laboratory Apparatus Pty Ltd., Narangba, QLD, Australia).

Respiration was measured twice over 2 days in five microcosms for each treatment using a procedure detailed by Bonner et al. [31]. Briefly, agar gel was used to set cresol red indicator solution [41, 42] in a breakable 96-well plate and the individual wells were placed for 2 h inside the microcosms, temporarily sealed with rubber stoppers as per Brackin et al. [39], after which the wells’ absorbance at 590 nm was read (Powerwave XS Spectrophotometer, Bio-Tek, USA). Microcosms were harvested after respiration was measured, by passing the soil through a 1.4-mm sieve for subsequent analysis of enzyme activity, functional profile and phospholipid fatty acids (PLFA). To estimate total hydrolytic enzyme activity (in five subsamples for each land use replicate), we used the fluorescein diacetate (FDA) colourimetric assay [43], which spectrophotometrically measures colour development resulting from hydrolysis of colourless FDA (by a very broad array of enzymes) into coloured fluorescein. Dividing enzyme activity by respiration calculates a type of enzyme efficiency (hydrolysis per C loss to respiration), an aspect of microbial efficiency that has been assessed in a variety of ways previously [31, 37, 44, 45].

Topsoil cores (0–10 cm) were collected from 10 locations per plot, passed through a 2-mm sieve, and analysed for total soil organic carbon (SOC) content using high temperature combustion (TruMac CN, LECO Corporation, St. Joseph, MI). Samples were first tested for presence of inorganic C (IC) using 1 M HCl, with any soil testing positive to presence of IC treated with H_2_SO_3_ prior to analysis of organic carbon content. Soil from the abovementioned microcosms was further sieved to a fine particle size (≤ 50 μm), representing the humus fraction proposed by Skjemstad et al. [46]. The procedure, outlined in Baldock et al. [47], disperses a 10-g sample using 5 g L^−1^ sodium hexametaphosphate solution, which is passed through a 50-μm sieve using an automated wet sieving system. The sample is lyophilised until completely dry, then finely ground using a Retsch MM400 Mixer Mill (RETSCH GmbH, Haan, Germany) to homogenize the sample. Total organic C content, δ^13^C, total N content and δ^15^N were estimated on the humus fraction using isotope ratio mass spectrometry (IRMS) [48]. Humus-fraction organic C (HOC) δ^13^C signatures allow estimation of HOC composition turnover under plantings, as lower values at a given age correspond to a faster replacement of C_4_ pasture-C with C_3_ tree-C [33]. Coarse (particulate) organic C (POC) was determined as the difference between the total organic carbon content of the ≤ 2 mm soil and HOC. Both POC and HOC fractions determined this way are liable in general to include a ‘resistant’, fire-derived fraction [47], but with the lack of fire history in the studied sites since plantation establishment, this fraction was unlikely to accumulate following reforestation and was not measured here. Soil bicarbonate-extractable P (‘Colwell-P’) was determined by method 9B2 of Rayment and Lyons [49].

The MicroResp system [42] was used to functionally profile the microbial communities. As this technique assesses respiratory responses to added substrates, which is just one aspect of catabolic activity, its capacity to characterize holistic soil microbial function is unknown, but it has proven an efficient method for catabolically discriminating distinct soil microbial communities. After adding circa 300 mg unsieved soil per well to deep-well microplates, 15 organic substrates (each dissolved in distilled water) and a distilled water control were added, each substrate to six wells for six technical replicates. Quantities of C and water added were kept consistent between substrates. Cresol red indicator set in agar gel, as summarized above, was used to estimate CO_2_ evolved during the 12-h following substrate additions. Sugars (glucose, fructose, sucrose), carboxylic acids (citric acid, α-keto-butyric acid), phenolic acids (vanillic acid, syringic acid), amino acids (phenylalanine, tryptophan, asparagine, glutamine, glycine), a dipeptide (glycine-phenylalanine), an amino sugar (glucosamine) and phytic acid dipotassium salt were chosen as compounds naturally occurring in soil that span a spectrum of lability and may be used variously as energy or nutrient sources.

Soil microbial composition was evaluated with PLFA analysis as per Bossio and Scow [50] on three pooled soil samples for each land use replicate. The capacity of this method to provide quantitative assessment of relative biomass of microbial groups made it preferable to genomic methods for the purposes of this study, where high taxonomic resolution was not a priority. Fatty acids thought to be of bacterial origin (i15:0, a15:0, 15:0, i16:0, 16:1ω7, i17:0, a17:0, cy17:0, 17:0, 18:1ω7 and cy19:0) were summed to calculate an index of bacterial biomass and 18:2ω6,9 provided an index of fungal biomass [51]. The fatty acids i15:0, a15:0, i16:0, i17:0 and a17:0 were used to estimate gram-positive bacterial biomass, and the fatty acids 16:1ω7, 18:1ω7, cy17:0 and cy19:0 were used to estimate gram-negative biomass [52]. The fatty acid 10me18:0 was used as an index of actinomycete biomass [53]. An index of microbial biomass was calculated as the sum of microbial PLFAs, which in turn allowed calculation of the quotient of respiration (qCO_2_, the respiration-to-biomass ratio), and the ratio of microbial biomass to soil organic C (C_mic_/C_org_), both of which have been used as metrics of soil microbial health [54, 55].

### Statistics

All statistical analyses were performed using R, version 3.2.4 (http://www.r-project.org/), with the packages ‘ggplot2’, ‘multcomp’, ‘car’, ‘plyr’, ‘MuMIn’ and ‘vegan’ [56–61].

To adjust for differences in baseline respiration in the MicroResp responses, values obtained from wells with only water added were subtracted from all other values, and resultant values were divided by the sum of responses for the sample [62]. Shannon’s diversity index was calculated from respiratory responses across the substrates using the equation *E* =  − ∑_*i*_*p*_*i*_*ln p*_*i*_, where *p*_*i*_ is the respiration induced by the *i*:th substrate expressed as a proportion of the sum of all respiration rates.

Linear mixed-effects models, Analysis of Variance and Tukey’s honest significance test were used for model fitting and testing. Optimal model selection was performed computationally using the ‘dredge’ function in the package ‘MuMIn’, which fits all possible models with all combinations of predictors and ranks them according to AICc (corrected Akaike Information Criterion). Only models passing diagnostic tests for heteroscedasticity, non-normality and outlier leverage were retained. Pairwise comparisons of treatments for significant differences were performed using Tukey’s honest significance test. Pearson’s product-moment correlation test was used to test suspected correlations between variables.

Responses of variables across land uses were assessed with linear mixed-effects models with ‘site’ as a random grouping variable. As the ‘planting’ land use was included in all models as intercept, errors for this land use were standard errors of the mean, while errors for the other two land uses were standard errors of the difference from the ‘planting’ land use. Using this approach, error bars of ‘pasture’ or ‘rainforest’ that do not overlap the mean value for ‘planting’ represent a significant difference of means.

The eight reforestation soils were not analysed as a chronosequence as there was, in our view, insufficient replication within age classes to overcome spatial variation by site. Considerably more of this background variation could be partitioned out by pairing each reforestation plot within site with adjacent baseline and (where applicable) old growth rainforest, and analysing land use differences with mixed effects models, treating the eight reforestation soils as replicates of a land use that averages 17 years in age.

The correlation between the matrices representing soil microbial function (MicroResp responses) and composition (PLFA values) was tested in 15-dimensional space directly using ordination into orthogonal axes followed by the Procrustes superimposition permutation test, which has been shown to have greater power and applicability than the Mantel test [63–65]. The choice of ordination was PCA (principal components analysis) after Chord transformation, because Chord distance is appropriate for this type of data, and Chord-transformed RDA (redundancy analysis) demonstrated the best explanatory power for constrained ordinations of this dataset [66] when compared with Hellinger-transformed RDA or CCA (canonical correspondence analysis). Chord-transformed RDA with MicroResp data as response and PLFA data as constraining axes followed by a permutation significance test was used to corroborate Procrustes results. Similarly, Chord-transformed data used in partial RDA ordinations, with ‘site’ as conditioning variable, allowed visualization of land use effects on microbial function and composition, and permutation tests on these ordinations allowed evaluation of statistical significance. All permutation tests were performed with 9999 permutations. Variance partitioning [67] was used for estimating relative soil organic C explanatory power of microbial function and composition combined (represented by matrices of the first four and five principal components from the abovementioned PCAs, respectively, together with enzyme efficiency), land use and site.

## Results

**Fig. 2:**
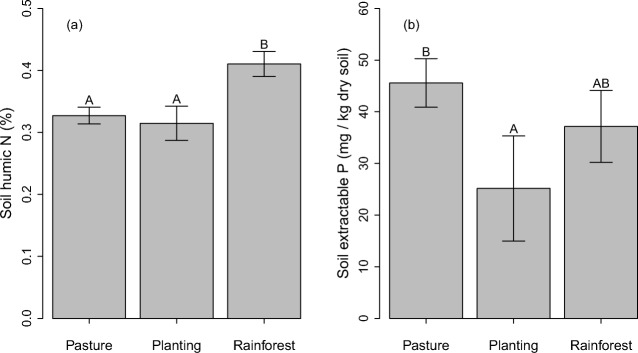
Soil **a** nitrogen in humic fraction (particle size ≤ 50 μm) and **b** Colwell-extractable phosphorous under pasture, mixed species plantings and reference rainforest sites in tropical north-eastern Australia. Error bars for plantings represent standard error of the mean, while errors bars for the two other land uses represent standard errors of difference from revegetation (see the ‘Statistics’ subsection of the ‘Materials and Methods’ section for more detail). Letters above bars represent Tukey’s significant differences

Soil organic C (SOC) content was highest in remnant rainforest, lowest in pasture and intermediate in plantings (Fig. 1a), which largely mirrored the pattern of particulate organic C (POC) (Fig. 1b). None of the SOC pools varied significantly with plantation age. Humic organic C (HOC) content did not significantly vary between soils from pasture and mixed species plantings, but it was significantly higher in reference rainforest soil (Fig. 1c). δ^13^C signatures of HOC revealed a significant turnover of HOC since establishment of plantings (Fig. 1d). Soil humic nitrogen (N) content followed the same pattern as HOC (Fig. 2a), while δ^15^N signatures of humic N was similar across land uses (data not shown). Extractable soil phosphorous was highest in pasture, intermediate in rainforest and lowest in plantings (Fig. 2b).

**Fig. 3:**
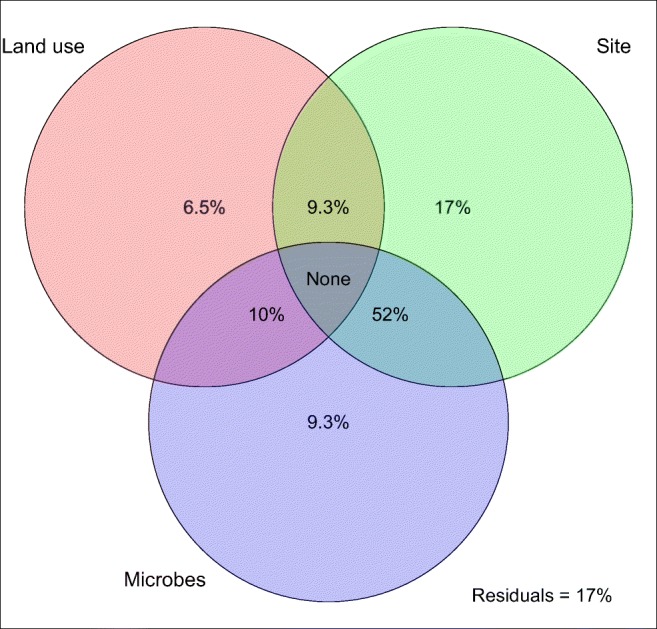
Variation partitioning Venn diagram depicting variation in soil humic organic C (HOC; organic C with particle size below 50 μm) explained by different land uses (old-growth forest, plantation, pasture; left circle), sites (sampling locations; right circle) and soil microbial function and composition combined (see earlier sections for detail; bottom circle). The sum of numbers in a circle represents the proportion of variation in HOC explained by that predictor. Numbers in overlapping areas represent variation explained by both predictors equally. Numbers outside of overlapping areas depict variation explained uniquely by a predictor. ‘Residuals’ represents variation not explained by any of the predictors. Note, the explained variance does not sum to 100% as an artefact of the variance partitioning algorithm inherent to this analysis

Variance partitioning allows evaluation of relative contributions of predictors to a response variable and indicated that most of the variation in HOC across sites and land uses could be also explained by microbial composition and function (microbial traits) (Fig. 3). Land use explained 25.8% of the variation in HOC, but of this only 6.5% was unique to this predictor, with the remaining 19.3% also explained by either site (9.3%) or microbial traits (10%). Similarly, 17% of the variation in HOC could be explained uniquely by site, whereas 52% of variation in HOC was explained equally by microbial traits and site, indicating substantial spatial variation in soil microbial traits associated with HOC.

**Fig. 4:**
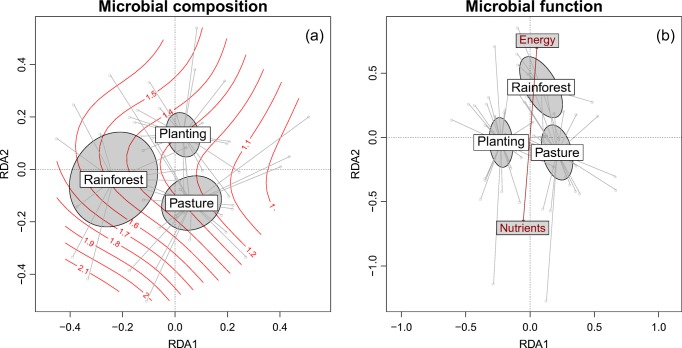
**a** Microbial composition (identified with PLFA analysis) and **b** microbial function (measured through substrate use and enzyme efficiency) as a function of three tropical land uses. See supplementary material for detailed PLFA and substrate use responses to land use. The axes are output from a Chord-transformed partial redundancy analysis (distance-based partial RDA using Chord distance), controlling for background variation across sites. Ellipses represent 95% confidence intervals for the mean. Contour lines in the left panel represent gram-positive to gram-negative bacterial biomass ratio, and arrows in the right panel represent directions of increase in responses to compounds containing nutrients (amino acids, a dipeptide, phytic acid and an amino sugar) and only energy (sugars, carboxylic acids and phenolic acids)

**Fig. 5:**
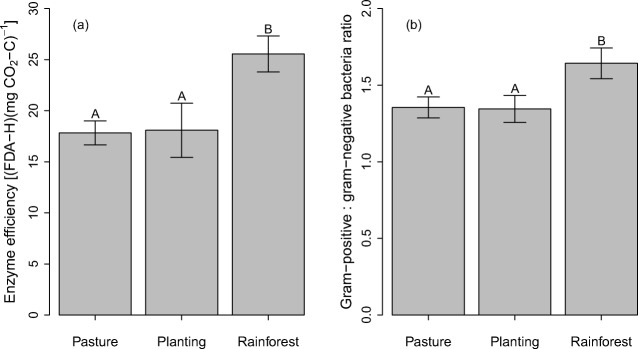
**a** Soil microbial enzyme efficiency and **b** bacterial gram-positive to gram-negative biomass ratio across three tropical land uses in north-eastern Australia. Enzyme efficiency is the quotient of fluorescein diacetate hydrolysis (FDA-H per hour, a measure of total hydrolytic enzyme activity) and respiration (mg CO_2_-C per hour), and the ratio of gram-positive and gram-negative bacteria is calculated as a ratio of PLFA markers for each bacterial group. Error bars for revegetation represent standard error of the mean, while error bars for the two other land uses represent standard errors of difference from revegetation (see the ‘Statistics’ subsection of the ‘Materials and Methods’ section for more detail). Letters above bars represent Tukey’s honest significant differences

Permutation tests of Chord-transformed partial redundancy analysis, controlling for random variation across sites, indicated that pasture, mixed species plantings and reference rainforest each had unique soil microbial composition (*P* < 0.001) and function (*P* < 0.001) (Fig. 5). Microbial function and composition were significantly correlated across soils under the three land uses (*P* < 0.001 from Procrustes superimposition test).

Although numerous rarefied microbial compositional and functional characteristics, including fungal/bacterial biomass ratio and Shannon’s functional diversity, were not detectably different across land uses, differences were observed with the ratio of gram-positive to gram-negative bacterial biomass, enzyme efficiency (microbial enzyme activity divided by respiration) (Fig. 5), and MicroResp responsiveness to compounds containing only energy (rather than those containing nutrients) (Fig. 4b). These indices showed the same signal as HOC, with significantly higher values in reference rainforest than in pastures and plantings, which in turn were indistinguishable (Figs. 4b, 5).

## Discussion

We compared soil organic C and microbial communities under rainforest restoration plantings with those under pasture, the land use preceding planting establishment and remnant rainforest, representing the theoretical endpoint of restoration. We found that stable (humic) SOC content (HOC) was unchanged after an average of 17 years since plantation establishment and not tracking towards recovery to pre-clearing levels. Soil microbial enzyme efficiency, a measure of how much C is retained versus lost to respiration during microbial activity, displayed the same pattern as HOC, being similar in pasture and plantings but significantly greater in rainforest soil. While overall soil microbial composition and function were significantly altered by plantings, the changes were not convergent on those observed in rainforest in the timeframe examined. The results do not support the hypothesis that plantings rapidly induce a carbon cycle characterized by high microbial efficiency and associated accumulation of stable SOC in the form of HOC. We conclude that in this study, diverse restoration plantings with over 20 native tree species do not successfully restore soil function within the first two decades.

The increase in total SOC with planting was entirely accounted for by changes in POC (particulate organic C). Because POC is considered a fast-turnover pool in contrast to the more stable HOC [46, 68], the SOC signal may be a poor reflection of long-term soil C sequestration outcomes with land use change. HOC δ^13^C signatures indicate that composition of HOC under plantings has changed, displaying an intermediate C_4_-C_3_ photosynthesis signal [69], yet the total pool size remains unchanged. This points to a set of soil characteristics constraining HOC pool size, and that those characteristics would need to be restored before stable SOM can be restored. In our study, more variation in HOC was explained by soil microbial function and composition than by land use. In particular, specific components of microbial function and composition—enzyme efficiency and the ratio of gram-positive to gram-negative bacteria—closely reflected the pattern of HOC as both were enduring at pasture levels in soil under plantings and substantially lower than in rainforest soils. These observations amount to a strong correlation between microbial traits and HOC, but causative associations between the two, if any, can only be speculated. If we speculate that the abovementioned constraint on HOC pool size is microbial, then recovery of soil fertility and structure through reforestation would depend in part upon restoration of the soil microbial community.

A coupling between microbial enzyme efficiency and HOC coheres with mechanistic theory. As HOC seems to a large extent to be composed of microbial necromass and residues [26, 29], higher microbial efficiency is predicted to allow larger inputs to HOC for a given quantity of plant C input. Shao et al. [70] observed increases in microbial lipids and necromass to predict future increases in SOC in a reforestation chronosequence. A mechanism for the ratio of gram-positive to gram-negative bacteria contributing to greater HOC is also readily available. Compiling data from 20 long-term field experiments, Schmidt et al. [71] estimated mean soil residence time for gram-positive bacterial residues to be approximately 45 years, more than twice that of gram-negative bacteria and even lignin, both of which average about 20 years, and similar to the mean residence time of bulk SOM. Gram-positive bacteria contain more peptidoglycan in their cell walls than gram-negative bacteria, a compound considered resistant to decomposition because of its complex structure and unusual amino acid composition [72, 73]. Peptidoglycan contains significant amounts of D-isomer amino acids, which are more stable in soil than their L-isomer counterparts [74]. Marine dissolved organic nitrogen appears to be largely derived from peptidoglycan, likely due to this stability [72]. Observations that stable SOM has a high content of amino compounds [75] may be partially explained by peptidoglycan and its stable isomers. Finally, we can speculate that a microbial community more inclined to invest in C acquisition rather than nutrient acquisition, as observed in our study under the old growth rainforest, may spare investment in degrading SOM (which has a lower C/N ratio) in favour of decomposing fresh plant residues (which have a higher C/N ratio).

Our results necessitate refinement of the working model of SOM formation. As stated above, the model contends that a high diversity of C substrates and minimal soil disturbance are likely to confer increases in SOM. These two conditions were met by the studied plantings. Indeed, aboveground native woody plant richness of local plantings typically reach values similar to reference forest within 25 years [5] and strict selection criteria in our study avoided sites with a history of tillage. Despite the theoretically favourable conditions, stable SOM did not measurably increase in the restoration plantings, and enzyme efficiency was seemingly unchanged relative to baseline pasture. It is possible that limited floristic compositional convergence in the early decades of reforestation [5, 12] may be a constraint to soil recovery [76]. For example, the restoration plantings, which tend to include N-fixing tree species [5], may have been in a phase of relative N-limitation sometimes demonstrated in tropical secondary succession [77], resulting in plant residues with low average C/N compared to old-growth rainforests. This substrate stoichiometric non-convergence may limit microbial convergence, with microbial composition and function recovering rapidly after forest N-limitation (and the associated abundance of N-fixers) has fallen to old-growth levels. If we speculate more generally that compositional recovery belowground and aboveground are linked [78], and SOM recovery is in turn tied to microbial recovery [29], then restoring aboveground biomass and plant species richness may not be sufficient to restore soil. Rather, aboveground compositional recovery may be a prerequisite for rapid soil restoration.

Microbial communities were not recovered in our study after almost two decades (on average) of diverse tree cover in the humid tropics, a biome in which 20 years can be considered a long rotation time for commercial plantations [79]. These findings are novel for tropical ecological restoration plantings (under which microbes have not to our knowledge been examined with a resolution beyond biomass and respiration), but similar to previous studies in temperate systems demonstrating that soil microbial characteristics show signals of previous land use decades after ecosystem restoration [22, 23]. Conceivably, recovery of the soil microbial community might be limited in at least two ways: (1) indirectly through deficiencies in the ecological conditions provided by plantings or (2) directly via barriers to dispersal that hinder assembly of a microbial community characteristic of reference forest.

Two types of interventions might be feasible if recovery of soil microbes is a prerequisite for soil restoration. These are conceptually analogous to *prebiotics* (beneficial substrates) and *probiotics* (beneficial microbes), used for manipulating mammalian gut systems. First, the reinstatement of ecological conditions (i.e. diverse litter substrates) that allow the desired microbial community to form through competitive forces might best be achieved by restoring aboveground composition of forest (i.e. full complement of plant species). Previous work in the study region and elsewhere shows that restoration plantings beget rapid recovery of aboveground structure and diversity, but sluggish compositional recovery [5, 12]. This is underpinned by slow recovery of a predictable set of recruiting plant species as well as overrepresentation of other species in plantings. These deficiencies and biases could be addressed through improved selection of species for initial planting or later enrichment. Second, direct inoculation with desired microbes to re-instate microbial community composition could be used to overcome barriers to dispersal. The effectiveness of soil microbial inoculation, much like that of probiotics, remains unresolved [80, 81]. Soil transplant and inoculation experiments aimed at accelerating microbial recovery have so far seen mixed results [78, 82–85], but such experiments remain rare. Both Wubs et al. [85] and van der Bij et al. [78] found that soil inoculations had strong effects on trajectories of plant community recovery, suggesting that potential feedback cycles between aboveground composition and belowground composition may further complicate restoration efforts. This is a direction of study warranting more research [86].

We focus on biological aspects of SOM recovery with reforestation, but physical and chemical aspects deserve attention. Soil texture and concentrations of reactive silicates, polyvalent cations and metal oxides and ions constrain SOM formation [26, 87, 88]. Although these factors are not strongly affected by land use change [89], they may furnish a soil with an upper limit of SOM concentration or alter the SOM response to litter quality [35, 88]. In our study, background variance in soil chemistry and physics was largely accounted for by site selection, but restoration efforts ideally need to consider them in advance of commencing intervention.

### Conclusions

Our study presents a well-controlled comparison of tropical soil responses to reforestation. We conclude that the reestablishment of aboveground structure through reforestation is unlikely to be sufficient for belowground ecosystem restoration within two decades, which in turn may be necessary for full recovery of many of the ecosystem services sought from forests, such as C sequestration. Strategies should be developed that accelerate soil microbial recovery as a priority in building a toolkit to achieve holistic forest ecosystem restoration. The apparent substantial time-lag for aboveground and belowground recovery following forest re-establishment further dissuades clearing of old-growth forest, as even high cost ecological restoration plantations are unlikely to wholly recover ecosystem functioning on a decadal timescale.

## Electronic supplementary material

ESM 1 (DOCX 601 kb)
