# Supplementary material for: Tropical Rainforest Restoration Plantations Are Slow to Restore the Soil Biological and Organic Carbon Characteristics of Old Growth Rainforest
Source: Microb Ecol. 2019 Aug 1;79(2):432–42. doi: 10.1007/s00248-019-01414-7 (PMC7033081; doi:10.1007/s00248-019-01414-7)
Supplement: Supplementary file 1 — (DOCX 601 kb) [file 248_2019_1414_MOESM1_ESM.docx]

**Supplementary material**

**

**

**Fig. S1.** Histogram of restoration plantation ages, with ticks across the x-axis representing individual sites.


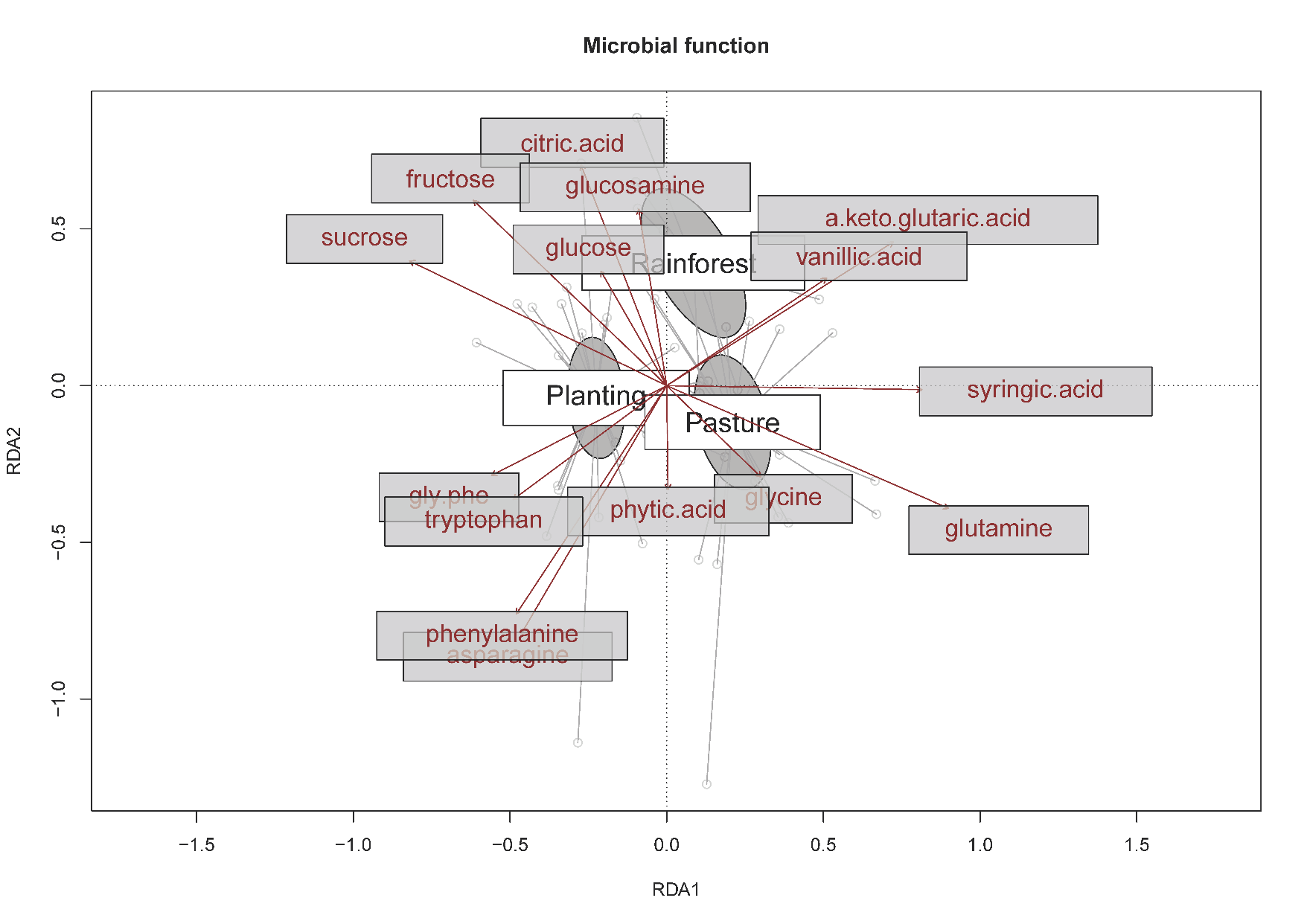


**Fig. S2.** Microbial function (measured through substrate use and enzyme efficiency) as a function of three tropical land uses. The axes are output from a Chord-transformed partial redundancy analysis (distance-based partial RDA using Chord distance), controlling for background variation across sites. Ellipses represent 95% confidence intervals for the mean. Arrows in right panel represent directions of increase in responses to various organic compounds.

**Table S1.** Substrate use profiles of soils from the three land uses in this study. The means were adjusted by subtracting water-induced respiration and expressing values as fractions of summed respiration across all substrates.

| Land use | Compound | Mean adjusted value | Standard error |
| --- | --- | --- | --- |
| Restoration planting | α.keto.butyric.acid | 0.057468677 | 0.00400254 |
| Restoration planting | asparagine | 0.130182667 | 0.009776076 |
| Restoration planting | citric.acid | 0.095028966 | 0.004910481 |
| Restoration planting | fructose | 0.110099274 | 0.003907902 |
| Restoration planting | glucosamine | 0.013339631 | 0.001652116 |
| Restoration planting | glucose | 0.103937521 | 0.007688383 |
| Restoration planting | glutamine | 0.098672575 | 0.00584793 |
| Restoration planting | gly.phe | 0.055985952 | 0.003162723 |
| Restoration planting | glycine | 0.039655173 | 0.003608675 |
| Restoration planting | phenylalanine | 0.047633505 | 0.003144678 |
| Restoration planting | phytic.acid | 0.040067194 | 0.002861386 |
| Restoration planting | sucrose | 0.105035109 | 0.004466602 |
| Restoration planting | syringic.acid | 0.022596747 | 0.002666613 |
| Restoration planting | tryptophan | 0.052127507 | 0.002208437 |
| Restoration planting | vanillic.acid | 0.028169503 | 0.002572919 |
| Pasture | α.keto.butyric.acid | 0.070942932 | 0.005361287 |
| Pasture | asparagine | 0.124611337 | 0.008911534 |
| Pasture | citric.acid | 0.087531185 | 0.008342977 |
| Pasture | fructose | 0.098286326 | 0.007585087 |
| Pasture | glucosamine | 0.013504943 | 0.002125017 |
| Pasture | glucose | 0.101630959 | 0.01053064 |
| Pasture | glutamine | 0.13194319 | 0.009717137 |
| Pasture | gly.phe | 0.04757193 | 0.002990846 |
| Pasture | glycine | 0.039917375 | 0.002741408 |
| Pasture | phenylalanine | 0.041111911 | 0.003948259 |
| Pasture | phytic.acid | 0.036334627 | 0.002287526 |
| Pasture | sucrose | 0.083973565 | 0.003481072 |
| Pasture | syringic.acid | 0.04240007 | 0.007190588 |
| Pasture | tryptophan | 0.046234605 | 0.002861719 |
| Pasture | vanillic.acid | 0.034005043 | 0.002480649 |
| Remnant rainforest | α.keto.butyric.acid | 0.098502508 | 0.013542445 |
| Remnant rainforest | asparagine | 0.093260501 | 0.010267778 |
| Remnant rainforest | citric.acid | 0.088909057 | 0.005137784 |
| Remnant rainforest | fructose | 0.106819345 | 0.011675114 |
| Remnant rainforest | glucosamine | 0.012077688 | 0.003558868 |
| Remnant rainforest | glucose | 0.11722491 | 0.010707535 |
| Remnant rainforest | glutamine | 0.096598346 | 0.005140532 |
| Remnant rainforest | gly.phe | 0.044158763 | 0.005446491 |
| Remnant rainforest | glycine | 0.03717212 | 0.003414755 |
| Remnant rainforest | phenylalanine | 0.031758014 | 0.002970571 |
| Remnant rainforest | phytic.acid | 0.043165832 | 0.009382156 |
| Remnant rainforest | sucrose | 0.11285627 | 0.00906906 |
| Remnant rainforest | syringic.acid | 0.039336828 | 0.002675871 |
| Remnant rainforest | tryptophan | 0.039805609 | 0.002818698 |
| Remnant rainforest | vanillic.acid | 0.038354208 | 0.003022022 |

**Table S2.** Phospholipid fatty acid profiles of soils from the three land uses in this study.

| Land use | Fatty acid | Mean (nmol PLFA / g soil) | Standard error |
| --- | --- | --- | --- |
| Restoration planting | X15.0.anteiso | 1.803362845 | 0.119147296 |
| Restoration planting | X15.0.iso | 3.394688822 | 0.191717316 |
| Restoration planting | X15.00 | 0.303403243 | 0.021482896 |
| Restoration planting | X16.0.iso | 2.111848544 | 0.130754192 |
| Restoration planting | X16.1.w5c | 1.258775814 | 0.092236803 |
| Restoration planting | X17.0.anteiso | 0.98893221 | 0.055600841 |
| Restoration planting | X17.0.cyclo | 0.718291624 | 0.04021704 |
| Restoration planting | X17.0.iso | 1.230069212 | 0.073886892 |
| Restoration planting | X17.00 | 0.230076696 | 0.015748805 |
| Restoration planting | X18.0.10.methyl | 1.043272088 | 0.073681064 |
| Restoration planting | X18.1.w7c.11.methyl | 0.415247326 | 0.029940722 |
| Restoration planting | X18.1.w9c | 2.496049499 | 0.202175412 |
| Restoration planting | X19.0.cyclo.w8c | 5.048674617 | 0.370130141 |
| Restoration planting | X16.1.w7c/16.1.w6c | 1.124755916 | 0.073815205 |
| Restoration planting | X18.2.w6 | 0.498361112 | 0.052814075 |
| Pasture | X15.0.anteiso | 1.693172022 | 0.153984819 |
| Pasture | X15.0.iso | 3.473298269 | 0.317231426 |
| Pasture | X15.00 | 0.310960146 | 0.023202669 |
| Pasture | X16.0.iso | 2.13500696 | 0.104424507 |
| Pasture | X16.1.w5c | 1.095321831 | 0.078138911 |
| Pasture | X17.0.anteiso | 0.91329456 | 0.050159491 |
| Pasture | X17.0.cyclo | 0.760909571 | 0.051667797 |
| Pasture | X17.0.iso | 1.193397692 | 0.080285071 |
| Pasture | X17.00 | 0.217366603 | 0.018988501 |
| Pasture | X18.0.10.methyl | 1.05223486 | 0.097156981 |
| Pasture | X18.1.w7c.11.methyl | 0.386944853 | 0.037838757 |
| Pasture | X18.1.w9c | 2.524163504 | 0.245659136 |
| Pasture | X19.0.cyclo.w8c | 5.616036603 | 0.803417903 |
| Pasture | X16.1.w7c/16.1.w6c | 1.091518962 | 0.077513761 |
| Pasture | X18.2.w6 | 0.475895698 | 0.044276485 |
| Remnant rainforest | X15.0.anteiso | 1.897869571 | 0.222362887 |
| Remnant rainforest | X15.0.iso | 3.416665789 | 0.278429015 |
| Remnant rainforest | X15.00 | 0.353538655 | 0.048373491 |
| Remnant rainforest | X16.0.iso | 2.225638914 | 0.164117162 |
| Remnant rainforest | X16.1.w5c | 1.197504765 | 0.114989794 |
| Remnant rainforest | X17.0.anteiso | 1.134057788 | 0.087863305 |
| Remnant rainforest | X17.0.cyclo | 0.747438895 | 0.085807209 |
| Remnant rainforest | X17.0.iso | 1.27664855 | 0.096558325 |
| Remnant rainforest | X17.00 | 0.264341065 | 0.032666918 |
| Remnant rainforest | X18.0.10.methyl | 1.278026859 | 0.19852083 |
| Remnant rainforest | X18.1.w7c.11.methyl | 0.404898691 | 0.069112988 |
| Remnant rainforest | X18.1.w9c | 3.186054941 | 0.66151113 |
| Remnant rainforest | X19.0.cyclo.w8c | 4.711231024 | 0.947838374 |
| Remnant rainforest | X16.1.w7c/16.1.w6c | 0.983899846 | 0.094706225 |
| Remnant rainforest | X18.2.w6 | 0.540740791 | 0.130830015 |
